# Origin of methane-rich natural gas at the West Pacific convergent plate boundary

**DOI:** 10.1038/s41598-017-15959-5

**Published:** 2017-11-15

**Authors:** Yuji Sano, Naoya Kinoshita, Takanori Kagoshima, Naoto Takahata, Susumu Sakata, Tomohiro Toki, Shinsuke Kawagucci, Amane Waseda, Tefang Lan, Hsinyi Wen, Ai-Ti Chen, Hsiaofen Lee, Tsanyao F. Yang, Guodong Zheng, Yama Tomonaga, Emilie Roulleau, Daniele L. Pinti

**Affiliations:** 1 Atmosphere and Ocean Research Institute, The University of Tokyo, Kashiwa, Japan; 2 Institute for Geo-Resources and Environment, National Institute of Advanced Industrial Science and Technology, Tsukuba, Japan; 3 Department of Chemistry, Biology and Marine Science, University of the Ryukyus, Okinawa, Japan; 4 Department of Subsurface Geobiological Analysis and Research, Japan Agency for Marine-Earth Science and Technology, Yokosuka, Japan; 5 Japan Petroleum Exploration Co., Ltd, Tokyo, Japan; 6 Department of Geosciences, National Taiwan University, Taipei, Taiwan; 7 Green Energy and Environment Research Laboratories, Industrial Technology Research Institute, Hsinchu, Taiwan; 8 Institute of Earth Sciences, Academia Sinica, Taipei, Taiwan; 9 Key Laboratory of Petroleum Resources, Gansu Province / Key Laboratory of Petroleum Resources Research, Institute of Geology and Geophysics, Chinese Academy of Sciences, Lanzhou, 730000 China; 10 Institute of Geological Sciences, University of Bern, Bern, Switzerland; 11 Laboratoire Magmas et Volcans, Université Clermont-Auvergne, CNRS - IRD, OPGC, 63178 Aubière, France; 12 GEOTOP & Départment des sciences de la Terre et de l′atmosphère, Université du Québec à Montréal, Montreal, Canada

## Abstract

Methane emission from the geosphere is generally characterized by a radiocarbon-free signature and might preserve information on the deep carbon cycle on Earth. Here we report a clear relationship between the origin of methane-rich natural gases and the geodynamic setting of the West Pacific convergent plate boundary. Natural gases in the frontal arc basin (South Kanto gas fields, Northeast Japan) show a typical microbial signature with light carbon isotopes, high CH_4_/C_2_H_6_ and CH_4_/^3^He ratios. In the Akita-Niigata region – which corresponds to the slope stretching from the volcanic-arc to the back-arc –a thermogenic signature characterize the gases, with prevalence of heavy carbon isotopes, low CH_4_/C_2_H_6_ and CH_4_/^3^He ratios. Natural gases from mud volcanoes in South Taiwan at the collision zone show heavy carbon isotopes, middle CH_4_/C_2_H_6_ ratios and low CH_4_/^3^He ratios. On the other hand, those from the Tokara Islands situated on the volcanic front of Southwest Japan show the heaviest carbon isotopes, middle CH_4_/C_2_H_6_ ratios and the lowest CH_4_/^3^He ratios. The observed geochemical signatures of natural gases are clearly explained by a mixing of microbial, thermogenic and abiotic methane. An increasing contribution of abiotic methane towards more tectonically active regions of the plate boundary is suggested.

## Introduction

Volatile elements are transported from the Earth’s interior to the hydrosphere and the atmosphere through volcanic and hydrothermal systems in addition to micro- and macro-seepages from active tectonic areas^1,2^. If the geological carbon cycle is well documented for its oxide forms, such as CO_2_^3–5^, much work is needed to describe the geological cycle of its reduced forms, such as methane (CH_4_)^6–8^. The shallower biological methane cycle is relatively well quantified, such as biological release from wetlands, rice paddies, animals and termites^9^ and its impact on the greenhouse gas budget of the atmosphere^10^. The deep geological methane cycle still has to be well constrained^8^. Much attention has recently focused on the sources of radiocarbon-free geological methane (i.e., older than 40kyrs) in the domain of petroleum geochemistry and microbiology^11–13^.

There are two types of geological methane; (a) abiotic methane originated in volcanic and geothermal systems^14–16^, and (b) methane derived from hydrocarbon generation processes in sedimentary basins^7^. In principle, the chemical and isotopic composition of this methane and the associated natural gases should correlate with the geodynamic setting of the region where these gases originate^17–20^.

**Figure 1:**
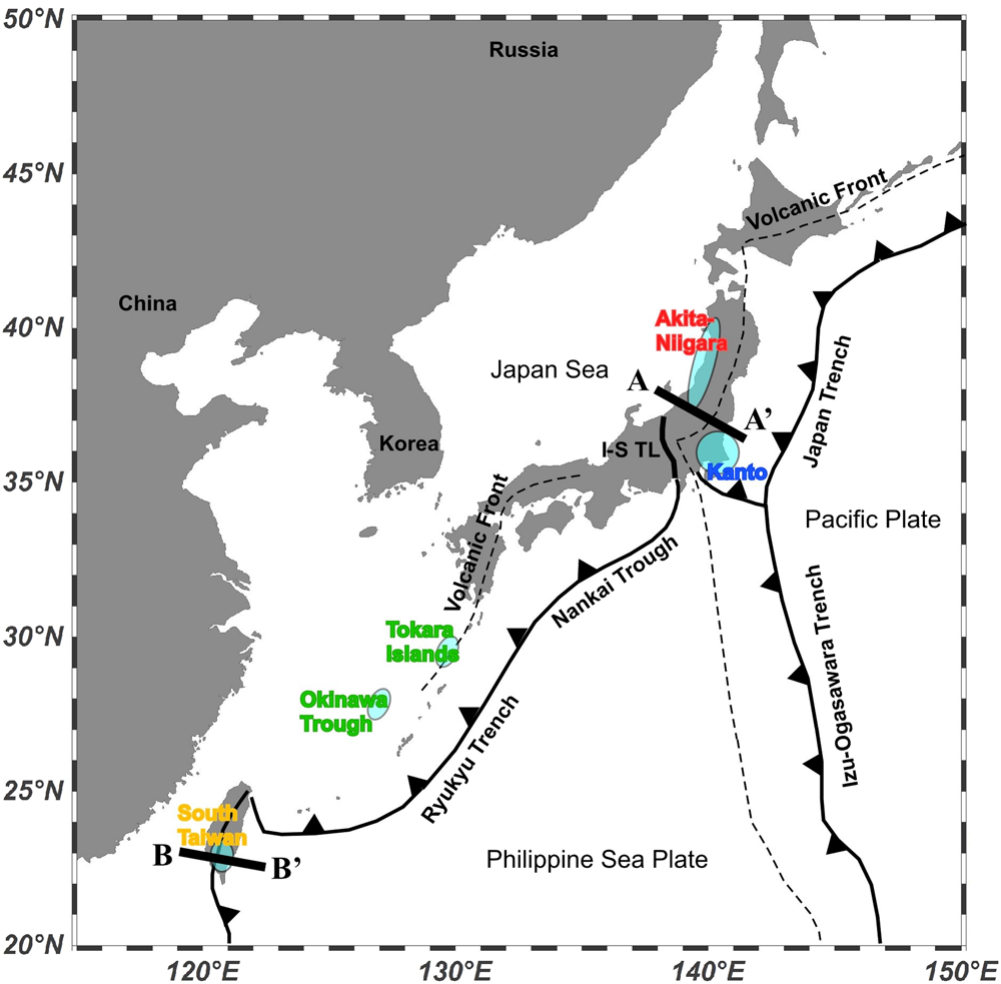
Sampling sites of methane-rich natural gases at the West Pacific convergent plate boundary with geotectonic settings. This figure was prepared using the Ocean Data View software^72^. A-A’ and B-B’ show cross sections of Fig. 5a and b, respectively.

In order to elucidate the origin of geological methane and its relationship with the geodynamic setting of the explored area, we collected natural gases in the West Pacific region (Fig. 1) and measured their chemical and isotopic compositions. This region is a window on a complete arc system with fore-arc, volcanic-arc and back-arc. Among various volatile elements in natural gases, helium-3 has been shown to be one of the most powerful tracers of fluid origin on Earth because of its mantle origin and since its isotopic variability depends on the geodynamic setting^21–25^, which is characterized by geophysical parameters such as terrestrial heat flow, Bouguer gravity anomaly, seismic velocity (P-wave perturbation), crustal deformation and magnetic anomaly. Therefore, the CH_4_/^3^He ratios of natural gases are expected to provide constraints on the origin of methane. In addition, we measured the nitrogen isotope composition of the sampled natural gases, which may provide further insights about their formation.

## Results

### Chemical compositions of methane-rich natural gases

Chemical compositions of 21 natural gas samples collected and analyzed in this work are listed in Supplementary Table S1 together with those from the literature^26,27^. Except for three CO_2_-rich samples in South Taiwan (CL-1, 2, 3), natural gas samples are dominated by hydrocarbon species such as methane and ethane (C_2_H_6_). Akita-Niigata natural gas samples show appreciable amounts of ethane and propane (C_3_H_8_), while the samples from the South Kanto gas field are dominated by an almost pure methane phase. Nitrogen is a minor component. South Taiwan samples have intermediate characteristics compared to the samples from the Akita-Niigata and South Kanto gas fields. Nitrogen content varies significantly from 0.1% to 21%, while oxygen abundances are generally close to 0.1%, suggesting that air contamination is negligible. All argon contents are much lower than the air value of 0.934%, while helium abundances are ranging from 2.7 ppm to 285 ppm, mostly higher than air value of 5.24 ppm, confirming an elemental signature that differs from atmospheric noble gas.

### Isotopic compositions of methane-rich natural gases

Isotopic compositions of carbon (^13^C/^12^C) in methane, nitrogen (^15^N/^14^N), helium (^3^He/^4^He) and argon (^40^Ar/^36^Ar) of the acquired natural gas samples are listed in Supplementary Table S2 together with the elemental ratios of ^4^He/^20^Ne, N_2_/^36^Ar, CH_4_/^3^He and CH_4_/(C_2_H_6_ + C_3_H_8_). C_1_, C_2_ and C_3_ in figures and tables correspond to CH_4_, C_2_H_6_ and C_3_H_8_, respectively. Supplementary Table S2 also reports representative data from the literature^26,27^. Carbon and nitrogen isotopes are expressed in the delta (δ) notation, as parts per thousand deviation (per mil, ‰) from the international standard PDB and atmospheric nitrogen, respectively. Helium isotopes are given in the Ra notation, where Ra is the atmospheric ^3^He/^4^He ratio of 1.382 × 10^−6^ (ref.^28^). The δ^13^C values of methane in this work vary significantly from −69.6‰ to −35.9‰. This range of values is consistent with previous work in the area^26,27^. Generally Akita-Niigata natural gas samples have δ^13^C values consistent with those of South Taiwan, both showing carbon isotope signatures being heavier than those determined in the South Kanto samples. δ^15^N values are ranging from −4.2‰ to + 2.8‰ in this work, indicating minor fractionation with respect to the air value of 0‰. There is no apparent discrepancy of the nitrogen isotope signature among Akita-Niigata, South Kanto and South Taiwan natural gases. Helium isotope ratios vary significantly from 0.06Ra to 6.29Ra (i.e., up to two orders of magnitude different values). The lowest value of 0.06 ± 0.05Ra is typical for crustal radiogenic helium, while the highest of 6.29 ± 0.14Ra indicates the presence of He with a typical subduction-like magmatic signature of 7.4 ± 1.3Ra^23^. Generally, the ^3^He/^4^He ratios of the South Kano samples are lower than the atmospheric air value of 1Ra, while those of Akita-Niigata show ratios higher than this value, indicating the occurrence of different proportions of mantle helium. South Taiwan samples are variable compared to those of South Kanto and Akita-Niigata. The northernmost samples (CL-1, 2, 3, and LS) show higher ^3^He/^4^He ratios than those in south, probable due to a mostly pure mantle source for gases in the northern Taiwan^29–31^. Most of the measured argon isotope ratios are similar to the air value of 298.6 ^28^ within the experimental error. A few samples from South Taiwan (CL-2 and WSD) show excess radiogenic ^40^Ar* with the ^40^Ar/^36^Ar ratios higher than air, but these ratios are much smaller than those observed in natural gases of geotectonically stable regions^32,33^.

## Discussion

Methane and heavier alkanes are the major components of the natural gases analyzed in this study. At first we consider the possible origin of these gases in relationship with the tectonic setting of the investigated gas fields at the West Pacific convergent plate boundary. In order to have a more comprehensive picture of the sources of light hydrocarbons in this area of the Pacific, we added recently published data collected from a shallow submarine hydrothermal system around the Tokara Islands^34^ (see Fig. 1). These samples were acquired right above the crater of a submarine volcano, using the acoustic survey system of the research vessel. Their chemical and isotopic compositions were measured in the same way than those of this work, so a direct comparison is allowed. The Tokara Islands are located just on the volcanic front between the Kyushu and Okinawa regions where Philippine Sea Plate is subducting beneath the Eurasian Plate (Fig. 1). Therefore, Tokara samples are tectonically different from Akita-Niigata (between volcanic arc and back-arc basin), South Kanto (frontal arc basin) and South Taiwan (collision zone) samples.

**Figure 2:**
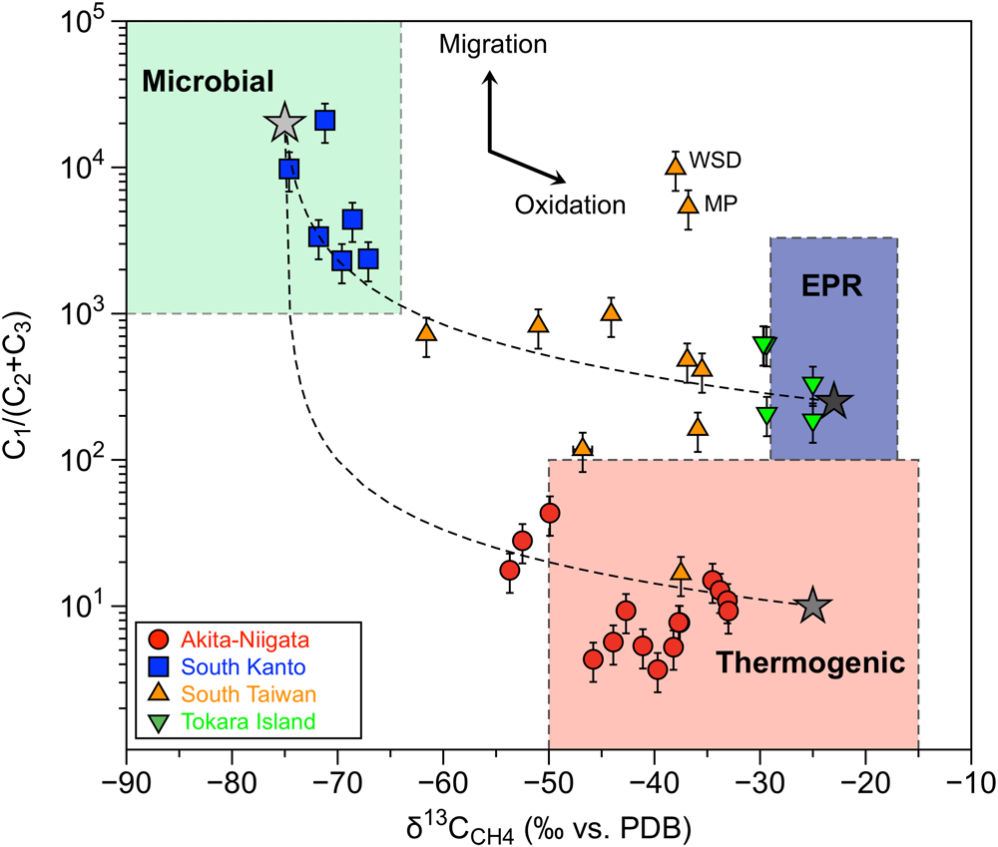
Correlation diagram (Bernard’s plot) between δ^13^C values and CH_4_/(C_2_H_6_ + C_3_H_8_) ratios of methane-rich natural gases at the West Pacific convergent plate boundary. Dotted curves show mixing lines between thermogenic and microbial end members, and between EPR and microbial end members of hydrocarbons. EPR indicates abiotic methane in hydrothermal systems of the East Pacific Rise. Arrows indicate the evolution of δ^13^C values and CH_4_/(C_2_H_6_ + C_3_H_8_) ratios during methane oxidation and migration, respectively.

Geological methane is classified into two groups according to its origin: abiotic origin related to magma and biogenic origin from sedimentary organic matter^8,14–16^. Furthermore, biogenic methane is divided into two subgroups based on the main processes responsible for its formation: thermogenic and microbial methane^35^. This classification was derived by combining δ^13^C values of methane and the CH_4_/(C_2_H_6_ + C_3_H_8_) ratio, in the so-called Bernard plot. Figure 2 shows the Bernard plot for methane-rich natural gases from this work together with the Tokara samples and two areas representing the isotopic and elemental values expected for the thermogenic and microbial methane end-members. Thermogenic methane is formed from the thermal cracking of kerogen or crude oil in sedimentary basins and it is characterized by heavy δ^13^C values higher than −50‰ and CH_4_/(C_2_H_6_ + C_3_H_8_) ratios lower than 100. On the other hand, microbial methane is produced at temperatures lower than 50 °C in shallower environments such as wetlands, by methanogenic archaea. Methanogenesis consists either of CO_2_ reduction or fermentation of methylated substrates^36^. Microbial methane has lighter δ^13^C values from −60‰ down to −110‰ and CH_4_/(C_2_H_6_ + C_3_H_8_) ratios higher than 1000.

It is noted that all South Kanto samples are located within the area of microbial origin, while most Akita-Niigata samples are of thermogenic origin except for two samples (Nos 16 and 17) with lighter δ^13^C values (Fig. 2). Therefore, South Kanto and Akita-Niigata samples are well explained by classical processes of natural gas generation^35^. On the other hand, it is difficult to explain the origin of the South Taiwan and Tokara samples by a simple binary mixing between thermogenic and microbial sources. For these outliers, either oxidation of microbial methane with a positive δ^13^C shift or molecular fractionation during migration of thermogenic methane is proposed^37–39^. In a Bernard plot, methane-rich natural gases from South Taiwan samples are located in the same region as that representing the methane in mud volcanoes worldwide^40^ (see Fig. 2). Etiope *et al*.^40^ claimed that the most suitable explanation is molecular fractionation due to fluid migration. Isotopic fractionation of carbon was generally not significant (i.e. always less than 5‰). In agreement with these authors, we suggest that methane in the South Taiwan samples is of thermogenic origin with some secondary modification by molecular fractionation. Even though Hong *et al*.^41^ carried out an intense sampling to obtain a mapping of the methane flux in the region, molecular fractionation was not taken into account as a possible process affecting the isotopic composition of methane in the area.

Tokara samples show heavier δ^13^C values than those of South Taiwan (Fig. 2). Based on the compilation of data from mud volcanoes worldwide, gas samples with δ^13^C values heavier than −30‰ are extremely rare: only one case of 201 sampled mud volcanoes showed similar values^40^. Hence, it is difficult to explain the origin of Tokara samples considering only a thermogenic origin. To infer the origin of the Tokara samples an additional third end-member, which represents gases of abiotic origin must be added in Fig. 2. The respective value ranges are deduced from findings in several submarine hydrothermal systems of the EPR (East Pacific Rise) from 18°S to 21°N^42,43^. Abiotic methane is characterized by δ^13^C values between −17‰ and −29‰ and CH_4_/(C_2_H_6_ + C_3_H_8_) ratios between 100 and 3200 as compiled from data obtained in several submarine hydrothermal systems of the EPR^42,43^ and those compiled by Sano and Fischer^23^. Tokara samples are well overlapping with the EPR region in Fig. 2 within experimental error margin, suggesting their abiotic origin in relation to the observed submarine volcanic activity^34^. Therefore, based on the above arguments and the geodynamic setting of the sampling sites characterized by parameters such as heat flow, gravity anomaly and seismic velocity, the order of contribution of abiotic methane is estimated as follows: Tokara > South Taiwan = Akita-Niigata > South Kanto. This relation is comparable to the one set by the measured helium isotope ratios: the Tokara samples (up to 4Ra) show much higher ^3^He/^4^He values than the South Kanto samples (<0.3 Ra), while those of the South Taiwan and Akita-Niigata samples are variable (Supplementary Table S2).

**Figure 3:**
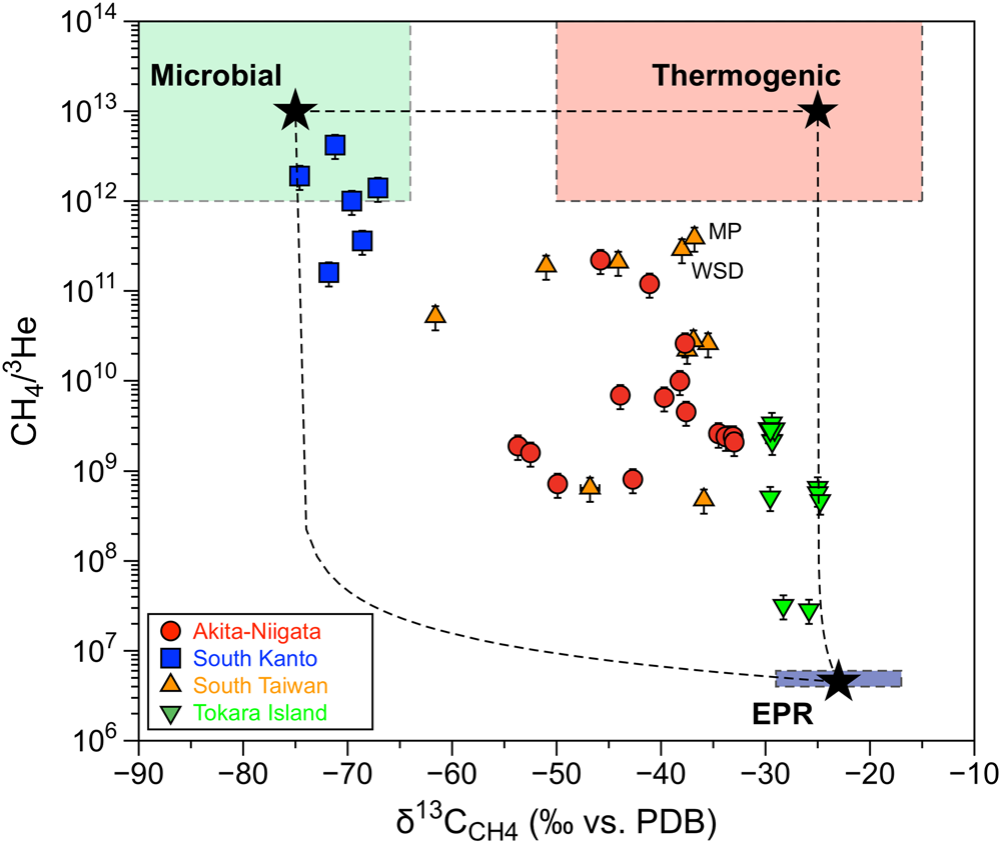
Correlation diagram between δ^13^C values and CH_4_/^3^He ratios of methane-rich natural gases. Dotted curves indicate mixing lines between EPR and thermogenic, and between EPR and microbial. All samples are located within the area delimited by the three end-members.

Even though abiotic (EPR-type) methane is discriminated from thermogenic and microbial methane in the Bernard plot, it is difficult to calculate quantitatively the abiotic contribution because the mixing line is complicated by the location of each end member. We need another parameter to discriminate the abiotic component in a more straightforward way than the CH_4_/(C_2_H_6_ + C_3_H_8_) ratio. The CH_4_/^3^He ratio is a possible candidate, since ^3^He traces univocally mantle contribution to fluids^22,23,44,45^. Since the CO_2_/^3^He ratio was successfully adopted as discriminant ratio for volcanic gases from island arcs worldwide^44^, the CH_4_/^3^He ratio may be also useful. Figure 3 shows the relationship between δ^13^C values of CH_4_ and the CH_4_/^3^He ratios. There are three independent end members in Fig. 3; EPR-type, thermogenic and microbial methane. It is plausible to assume that the average molecular ratio of CH_4_ to ^3^He in an EPR hydrothermal system, 5 × 10^−6^ (ref.^20^), is representative of abiotic methane derived from mid-ocean ridges associated to a δ^13^C value of −23 ± 6‰^23,42,43^. On the other hand, the CH_4_/^3^He ratio of the biogenic and the crustal end members was estimated to be 5 × 10^12^ by Wakita *et al*.^26^ and 1 × 10^13^ by Sakata *et al*.^27^. These values are very similar to the CO_2_/^3^He ratio of 1 × 10^13^ in limestone and sedimentary end members when Sano and Marty^46^ discussed the origin of carbon in fumarolic gases from island arcs. In this work, we assume that both microbial (i.e., biogenic) and thermogenic (i.e., crustal) methane have the CH_4_/^3^He ratio of 1 × 10^13^. We take a δ^13^C value of −25 ± 5‰ for the thermogenic end-member based on the most mature gas in the Akita-Niigata region^47^ and methane in submarine sediment-associated system^11,48^. On the other hand, we adopted a microbial δ^13^C end member of −75 ± 5‰ from Wakita *et al*.^26^.

All data including Tokara submarine samples are distributed within the mixing area of the three end members: EPR-type, thermogenic, and microbial methane (Fig. 3), suggesting that the mixing hypothesis is valid. Therefore, it is possible to deconvolute each contribution using the following equations:

1 $${{\rm{\delta }}}^{13}{{\rm{C}}}_{{\rm{Meas}}}={{\rm{\delta }}}^{13}{{\rm{C}}}_{{\rm{EPR}}}\times E+{{\rm{\delta }}}^{13}{{\rm{C}}}_{{\rm{Ther}}}\times T+{{\rm{\delta }}}^{13}{{\rm{C}}}_{{\rm{Micr}}}\times M$$

2 $${1/(C{H}_{4}/{}^{3}{\rm{H}}{\rm{e}})}_{{\rm{Meas}}}=E/{({{\rm{CH}}}_{4}/{}^{3}{\rm{H}}{\rm{e}})}_{{\rm{EPR}}}+T/{({{\rm{CH}}}_{4}/{}^{3}{\rm{H}}{\rm{e}})}_{{\rm{Ther}}}+M/{({{\rm{CH}}}_{4}/{}^{3}{\rm{H}}{\rm{e}})}_{{\rm{Micr}}}$$

with

3 $$E+T+M=1$$

where subscripts *Meas*, *EPR*, *Ther* and *Micr* refer to measured methane values, EPR-type abiotic methane, thermogenic methane, and microbial methane, respectively. With δ^13^C_EPR_ = −23 ± 6‰, δ^13^C_Ther_ = −25 ± 5‰, δ^13^C_Micr_ = −75 ± 5‰, (CH_4_/^3^He)_EPR_ = 5 × 10^6^, (CH_4_/^3^He)_Ther_ = 1 × 10^13^ and (CH_4_/^3^He)_Micr_ = 1 × 10^13^, we can calculate the contributions of the three components *E* (abiogenic), *T* (thermogenic), and *M* (microbial) in the samples. The deconvolution results of the lower and upper ranges at each site are listed in Supplementary Table S3 together with their end members. In this calculation, the molecular fractionation observed in the CH_4_/^3^He ratios and the isotopic fractionation indicated by the δ^13^C values during the fluid migration from the gas source to the sampling site are not taken into account. Etiope *et al*.^40^ suggested that isotopic fractionation resulting in higher δ^13^C values in mud volcano samples is less than 5‰ due to a segregation process or a chromatographic effect during fluid migration towards the planet’s surface. On the other hand, the molecular ratio CH_4_/(C_2_H_6_ + C_3_H_8_) of some mud volcano samples may increase two orders of magnitude during advective fluid migration^40^. If this is the case, the original CH_4_/^3^He ratios may be altered by the migration from the gas source to the sampling site. There are two samples in South Taiwan (WSD and MP) characterized by high CH_4_/(C_2_H_6_ + C_3_H_8_) ratios of 5000 ~ 10000 with thermogenic δ^13^C values of −36‰ ~ −38‰ (Fig. 2). Their CH_4_/^3^He ratios show the maximum values of 3 ~ 4 × 10^11^ among the South Taiwan samples and a correction considering the effects of fluid migration is expected to result in 100 times smaller values. If so, the contribution of EPR-type methane would be 100 times larger. Yet the corrected values (0.12% ~ 0.17%) remain smaller than the average EPR-type methane contribution of the South Taiwan samples (0.34%; Supplementary Table S3). Note that the northernmost samples (CL-1, 2, and 3) show the higher EPR-type contribution between 0.8% and 1.8% than other samples, suggesting the transition signature from collision in South to subduction in North Taiwan^29–31^. This could be verified in future by intensive sampling and isotopic analysis of gases from mud volcano samples.

Based on the deconvolution equations, the microbial methane contribution in the South Kanto samples reaches the maximum average value of 91%, while EPR-type methane is only 0.0008%, that is, negligibly small (Supplementary Table S3). This is consistent with the location of the South Kanto gas field in a non-volcanic frontal arc region with low terrestrial heat flow^49^ and at least 100 km far from active volcanoes^17^. The respective water-soluble natural gases are of typical shallow origin, i.e. produced by microbial activity. On the other hand, the thermogenic methane contribution in the Akita-Niigata samples is larger than 50%, which is most likely derived from a reservoir in volcaniclastic rock formations resulting from submarine magmatic activities in the middle Miocene^26^. This methane was possibly produced by cracking of kerogen and/or petroleum together with appreciable amounts of microbial methane^27^. The discrepancy in the abiotic methane contribution in this study and the one inferred by Wakita *et al*.^26^ is attributable to the choice of the EPR-type end member. We took the average of the measured CH_4_/^3^He ratios of hydrothermal fluids in EPR from 18°S to 21°N^42,43^, while Wakita *et al*.^26^ assumed a total carbon to ^3^He ratio of 2 × 10^9^ in the fluids and vesicles of mid-ocean ridge basalt glasses^1^. The total carbon consists of 99% CO_2_ and the CH_4_ contribution is less than 1% in EPR-type fluids. Wakita *et al*.^26^ considered that all hydrothermal CO_2_ was converted into CH_4_ by the Fischer-Tropsch reaction, which was possible in presence of abundant hydrogen. Considering the hydrogen content of modern EPR fluids being less than 1% of total gas composition^42,43^, it is difficult to justify the occurrence of Fischer-Tropsch reactions in the investigated hydrothermal system. Therefore, we decided to adopt the average CH_4_/^3^He ratio of 5 × 10^6^ for the EPR-type end member as a minimum.

Natural gases from mud volcanoes in South Taiwan show a thermogenic contribution of approximately 65% and a microbial share of 35%, which is very similar to those of Akita-Niigata samples (Supplementary Table S3), although the tectonic settings are different. Sun *et al*.^50^ reported geochemical analyses of natural gases from 17 mud volcanoes in South Taiwan. Consistently with our data, their δ^13^C values of methane varied from −26.5‰ to −58.0‰. Sun *et al*.^42^ also measured the δ^13^C values of mud volcano sediments. Their data did not support a direct relation between the emitted natural gases and the sediments in terms of production by the break-up of the organic matter, but suggested a deeper origin of gases, migrated upward through active faults in the region. Nevertheless, the authors concluded that natural gases are derived from microbial to thermally over-matured sources, which is consistent with our estimates based on the δ^13^C values and CH_4_/^3^He ratios. The northernmost samples (CL-1 and 2) with a high EPR-type contribution are also characterized by thermogenic proportions of 50–70% of the total, suggesting heat source in crust.

Finally, we discuss the possible origin of the Tokara hydrothermal gases. If the deconvolution of these methane gases is valid, they are almost of thermogenic origin (91% on average) with a contribution of EPR-type abiotic methane of only 3.2% of the total (Supplementary Table S3). However, the seawater samples were collected immediately above the crater of the emitting shallow submarine volcano and the *in situ* contribution from marine sediments might be negligibly small^34^. There is no sedimentary layer in the region and it is difficult to find thermogenic source in the hydrothermal system. On the other hand, Sano and Marty^38^ reported that up to 20% of the carbon in high-temperature volcanic gases in subduction zones is derived from a mid-ocean ridge basalt-type source, while the major fraction is attributable to carbon derived from subducted marine carbonates. They also suggested that subducted marine sediments should contribute an appreciable amount, approximately 10%, in volcanic gases. The Tokara Islands are located in the volcanic front of the Ryukyu arc. The subducted sediments of the descending Philippine Sea Plate may be involved in the arc magma, i.e., becoming a possible source of thermogenic methane. In summary, according to our quantitative estimate of EPR-type methane contributions, we can estimate the order the sampling sites with respect to the contribution of abiotic methane as follows: Tokara > South Taiwan ≥ Akita-Niigata > South Kanto. This is in line with the estimates by using the Bernard plot in the previous section.

Nitrogen is a minor component of methane-rich natural gases and commonly exists in petroleum worldwide^51^ and is considered as common contaminant in oil industry. Since the pioneer work on nitrogen isotopes in natural gases by Hoering and Moore^52^, the number of published works on the subject remains significantly smaller than those of carbon isotopes in methane-rich gases^53–57^. The major source of nitrogen in natural gases has been attributed to the thermal breakdown of organic sediments at depth^58^, which may occur in combination with thermogenic methane production. In addition, microbial decomposition may liberate organic-bound nitrogen at shallow depth^59^. This nitrogen is generally characterized by positive δ^15^N values from + 3‰ to + 10‰^60^. On the other hand, nitrogen in mid-ocean ridge basalt glasses and peridotitic diamonds has negative δ^15^N values of −4 ± 1‰ and −6.5 ± 1.5‰, respectively^61,62^. Based on these observations, upper-mantle nitrogen was characterized by a δ^15^N value of −5 ± 2‰^63^. Atmospheric nitrogen could be another source in natural gases with a N_2_/Ar ratio between 38 and 84^57^. Thus, the presence of nitrogen in natural gases can be explained by mixing of three end-members: the mantle, the sediments, and the atmosphere.

Nitrogen isotopes of high-temperature volcanic gases in island arcs were measured and compiled by Sano *et al*.^4^. The δ^15^N values varied from 0.1‰ to 4.6‰, i.e. similar to atmospheric nitrogen with a small positive shift. It was difficult to distinguish the source based on the nitrogen isotopes only, as the contributing end members could have been either atmospheric nitrogen with δ^15^N = 0‰ or a mixture of sedimentary nitrogen with δ^15^N = + 7 ± 4‰ and upper mantle nitrogen with δ^15^N = −5 ± 2‰. Sano *et al*.^63^ invented the method of deconvolution based on the N_2_/^36^Ar ratios together with δ^15^N values. Note that, compared to the total N_2_/Ar ratio, the N_2_/^36^Ar ratio is a better parameter to detect atmospheric contribution because the contribution of radiogenic ^40^Ar* might be significantly high in some natural gases of crustal origin^32,33^. We applied the deconvolution method by Sano *et al*.^63^ to our data. Nitrogen data of the Tokara samples have not been measured, as seawater samples are generally dominated by atmospheric nitrogen. Instead we refer to nitrogen data of submarine hydrothermal mineral deposits in the Okinawa Trough (Fig. 1). The gases were extracted from vesicles of a sulfide chimney and measured by the same analytical system used in the present work^64^.

**Figure 4:**
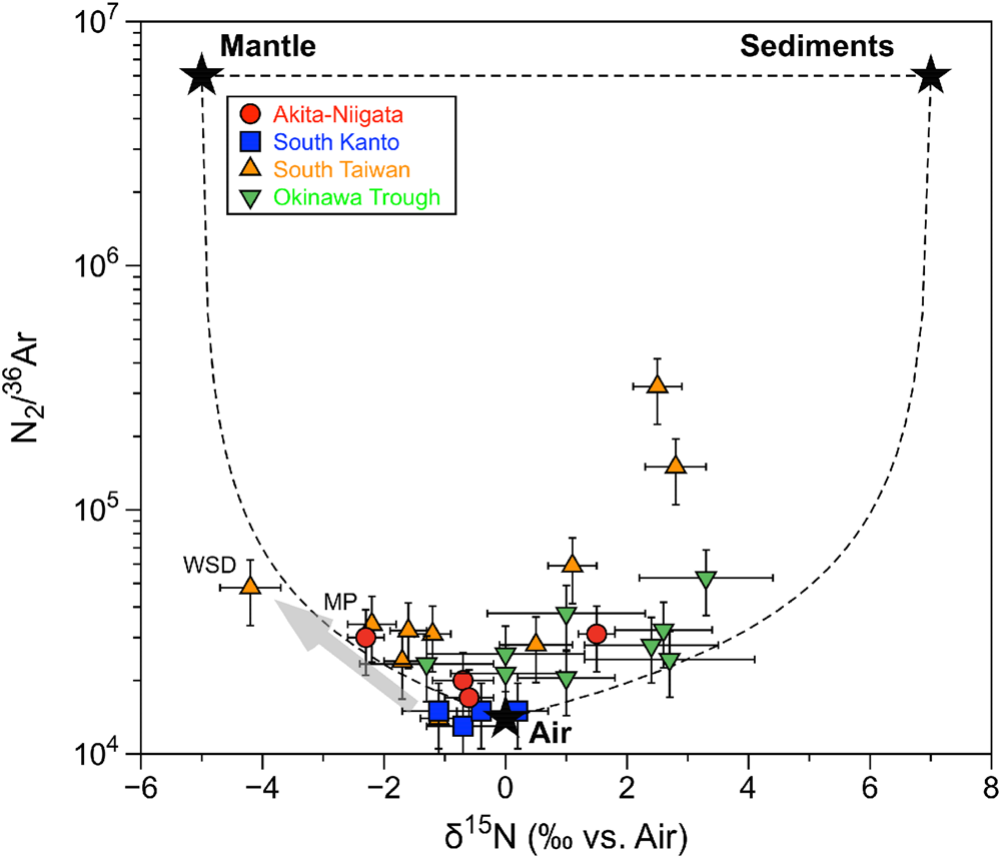
Correlation diagram between δ^15^N values and N_2_/^36^Ar ratios of methane-rich natural gases at the West Pacific convergent plate boundary. Dotted curves show mixing lines between Air + ASW and sediments, and between Air + ASW and mantle. ASW is an air saturated water at 20 °C. Arrow indicates fractionation effects due to fluid migration.

Figure 4 shows a relationship between δ^15^N values and N_2_/^36^Ar ratios of methane-rich natural gases in this study together with the Okinawa Trough samples. Most samples (with a few exceptions that include a mud volcano in South Taiwan, WSD) are distributed within the mixing area delimited by the three end members (i.e., mantle, sedimentary and atmospheric nitrogen) suggesting that the mixing hypothesis is likely valid. Hence, it is possible to deconvolve each contribution using the simple equations described by Sano *et al*.^63^. With the end members δ^15^N_Mantle_ = −5‰, δ^15^N_Sediment_ = + 7‰, δ^15^N_Air = _0‰, (N_2_/^36^Ar)_Mantle_ = 6 × 10^6^, (N_2_/^36^Ar)_Sediment_ = 6 × 10^6^, and (N_2_/^36^Ar)_Air_ = 1.4 × 10^4^ (this value is in between that of air and air-saturated water), we can calculate the percentage of the three components, i.e. mantle, sedimentary and atmospheric nitrogen, in the samples. In this calculation, the elemental fractionation of the N_2_/^36^Ar ratio and the nitrogen isotope fractionation from the gas source to the sampling site are not taken into account, which sometimes has a significant effect on methane-rich gases. Two mud-volcano samples (WSD and MP) of South Taiwan are probably affected by advective fluid migration resulting in high CH_4_/(C_2_H_6_ + C_3_H_8_) ratios as discussed in a previous section. Note that the WSD sample shows a very negative δ^15^N value, possibly generated by isotope fractionation due to migration. At the same time, the N_2_/^36^Ar ratios are expected to increase significantly because of the preferential enrichment in lighter atoms/molecules. Except for a couple of samples that seem to show a migration effect, the distribution of the natural gas data in Fig. 4 is not so much different compared to the one of the Okinawa Trough samples^64^. This distribution is also similar to the one observed for the Tatun Volcanic Group in North Taiwan^65^.

Based on the deconvolution calculation for nitrogen, air contribution in the South Kanto samples is dominant, approximately 94% on average. If we take into account the errors on the δ^15^N values and the N_2_/^36^Ar ratios, these data are consistent with the atmospheric composition, suggesting that actual contributions of other end members such as mantle and sediment are significantly smaller. This agrees well with the results of methane end-member deconvolution showing negligible abiotic contribution and may reflect the geotectonic setting characterized by a non-volcanic environment. On the other hand, the average mantle contributions to the Akita-Niigata and South Taiwan samples are 27% and 36%, respectively. The northernmost samples in South Taiwan (CL-1, 2 and LS) show the highest proportions of mantle nitrogen up to 45%. These estimates are larger than those obtained for high-temperature volcanic gases of island arcs^4^ that are probably affected by fractionation during the migration from the gas source to the sampling site. Due to the fluid migration process, δ^15^N values may decrease, while N_2_/^36^Ar ratios would increase (see an arrow in Fig. 4). These variations would make the apparent mantle contribution larger. Even though it is difficult to correct numerically this alteration, isotopic fractionation affecting the δ^15^N values of mud volcano samples may be less than 5‰ because the alteration of δ^13^C of methane is less than 5‰^32^ and the relative mass difference between ^15^N^14^N and ^14^N^14^N is smaller than between ^13^CH_4_ and ^12^CH_4_. Based on the deconvolution of the nitrogen sources, the order of contribution of mantle-derived nitrogen is as follows: South Taiwan ≥ Akita-Niigata > South Kanto. This relation is similar to the one inferred for the abiotic methane contribution in natural gases, probably reflecting a geotectonic setting where high heat flow, low seismic velocity and negative gravity anomaly prevails, while crustal deformation and magnetic anomaly are not related (Supplementary Table S4).

## Conclusion

**Figure 5:**
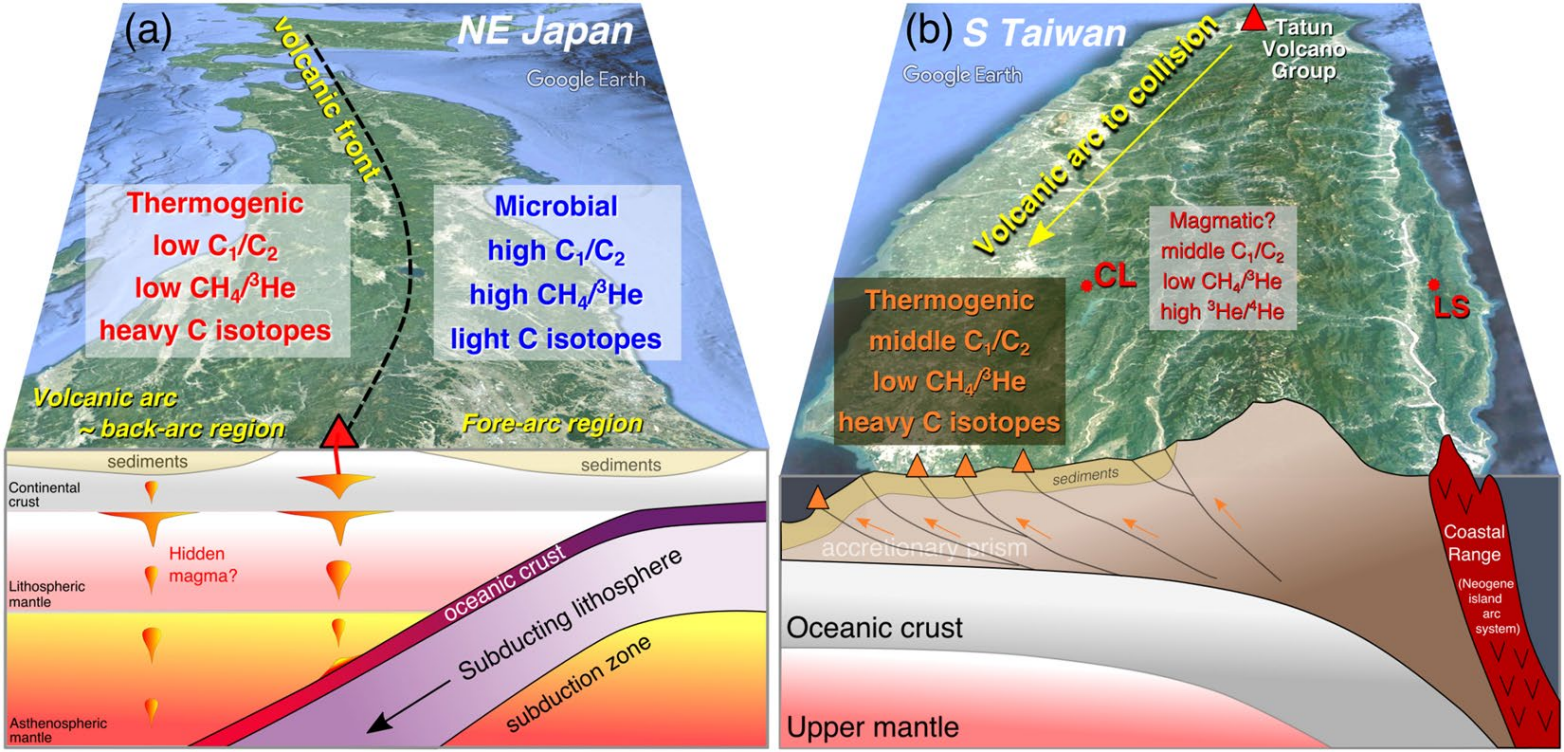
Schematic diagram showing a relationship between origin of methane-rich natural gas and geographical and geodynamic settings of the Western Pacific convergent plate boundary. (**a**) Vertical cross section of A-A’ in Fig. 1. In fore-arc region of Northeast Japan, there is no magma generated and methane is totally microbial, while in volcanic-arc ~ back-arc region, thermogenic methane may be attributable to hidden magma in crust. Map is generated by Google Earth Pro 7.3.0.3832 (64-bit) (Mac version). Original figure was created by Hsiaofen Lee and Tefang Lan. (**b**) Vertical cross section of B-B’ in Fig. 1. In South Taiwan, collision tectonics is distinguished where thermogenic methane is generated with mud volcanoes. There is a transition signature from subduction in north to collision in south Taiwan. CL samples may be affected by subduction-type volatiles. Original figure was created by Yves Rene Descatoire for Earth Observatory of Singapore^73^ and modified by Hsiaofen Lee and Tefang Lan.

We have measured the elemental and isotopic compositions of carbon, nitrogen, argon and helium in methane-rich natural gases from the West Pacific convergent plate boundary. A relationship between origin of methane-rich natural gas and geographical and geodynamic settings is summarized in Fig. 5. The South Kanto gas samples of Northeast Japan show light δ^13^C values, high CH_4_/(C_2_H_6_ + C_3_H_8_) and CH_4_/^3^He ratios, atmospheric δ^15^N values and N_2_/^36^Ar ratios, that are well explained by a microbial origin characterized by low-temperature processes and a non-volcanic signature typical of a fore-arc basin. The Akita-Niigata samples show heavy δ^13^C values, low CH_4_/(C_2_H_6_ + C_3_H_8_) and CH_4_/^3^He ratios, variable δ^15^N values and N_2_/^36^Ar ratios, suggesting a thermogenic origin possibly related to magmatic heat in a volcanic-arc region. The South Taiwan samples are characterized by heavy δ^13^C values, intermediate CH_4_/(C_2_H_6_ + C_3_H_8_) ratios, low CH_4_/^3^He ratios, variable δ^15^N values and N_2_/^36^Ar ratios, probably related to the collision tectonics with some magmatic heat and active fault. The largest contribution of abiotic methane is in South Taiwan samples with 0.34% on average. A slightly smaller average proportion of 0.21% is observed in the Akita-Niigata samples. The abiotic contribution in the South Kanto samples is negligibly small (i.e., only 0.0008%). These trends are consistent with the tectonic settings of the plate characterized by variations in heat flow, seismic velocity and gravity anomaly.

## Methods

### Sampling sites

Methane-rich natural gas samples were collected in three tectonically different regions in West Pacific convergent plate boundary (Fig. 1). The South Kanto natural gas field is located in the frontal arc region of Northeast Japan where Pacific Plate is subducting beneath Eurasia Plate, where there are no active volcanoes or hydrothermal systems at present. Natural gases accumulated in Pliocene-Pleistocene sedimentary rocks of the Kazusa Group, Japan^66^. The Kazusa Group reaches 2000–4000 m in thickness with several turbidite formations deposited in a deep basin, and it is located across a large area of the Tokyo Bay region. Four natural gas samples were collected from production wells at their wellheads. At the sampling site, a lead glass container with vacuum valves at both ends was filled with water to avoid air contamination, and one end was connected to the outlet of the well. After replacing the water in the container by collected gases, both valves were closed. The Akita-Niigata natural gas field is located in Northeast Japan, face to the Sea of Japan. The gas field lies on a basin developed in a transient slope from volcanic arc to back-arc region. The volcanic arc is attributable to magma generated by subduction of the Pacific Plate beneath the Eurasia Plate (Fig. 1). There are a few active volcanoes close to the region such as Mt. Chokai and Mt. Hijiori. Most natural gases accumulated in volcanoclastic rock formations, the so-called “Green Tuff” which may be attributable to large-scale magmatic activities of the middle Miocene^67^. Six natural gas samples were collected from production wells using the same method as described in the previous paragraph. Taiwan is located at the collision boundary between the Philippine Sea Plate and the Eurasia Plate and developed a typical accretionary prism in the southern part of the island. Several mud volcanoes are distributed in south Taiwan region, reflecting compressional tectonics^68^. They are exhaling significant amounts of methane-dominated gases from gas sources located in sandstone beds with mud stone matrix^69^. Eleven natural gas samples were collected using the previously described water replacement technique in active mud volcanoes and seepages of South Taiwan.

### Analysis

All measurements have been achieved at Atmosphere and Ocean Research Institute, the University of Tokyo. Major gas compositions were measured by a quadrupole mass spectrometer equipped with an electron multiplier detector (Prisma QMS200, Preiffer Ltd.). Abundances of CH_4_, heavier hydrocarbons, CO_2_, N_2_, O_2_, Ar, and He were determined by comparing peak heights of the sample with those of standard gases (the details are summarized in Sano *et al*.^63^). The overall error of the analyses was approximately 10% at 2σ for major gases such as CH_4_ and N_2_, which is estimated by the reproducibility of the standard measurements. The error may be up to 30% for trace gases like He and Ar. Carbon isotopes (^13^C/^12^C ratios) of CH_4_ were measured using a continuous flow GC-IRMS system (IsoPrime100 equipped with a vario-EA system; Isoprime Ltd.). The overall analytical error was approximately 0.3‰ at 2σ^70^. Nitrogen isotopes (^15^N/^14^N ratios) of N_2_ were measured by the same GC-IRMS system for samples with nitrogen abundances larger than 1%. For N_2_ abundances smaller than 1%, we used a nitrogen isotope mass spectrometer under static vacuum operation (VG3600; Micromass Ltd.). The ^15^N/^14^N ratio was measured at masses 28 and 29 on a single Faraday cup using magnetic field cycling after separation and purification of nitrogen^71^. Helium and neon were purified using charcoal traps and Ti getters in an all-metal ultra-high vacuum extraction line. After the ^4^He/^20^Ne ratio measurement using a quadrupole mass spectrometer, helium was separated from neon using a cryogenic charcoal trap and the ^3^He/^4^He ratio was measured by a helium isotope mass spectrometer (Helix SFT; GV Instruments Ltd.). The observed ^3^He/^4^He ratio was calibrated against standard atmospheric helium^28^. The ^40^Ar/^36^Ar ratios were measured by a quadrupole mass spectrometer (HAL201, Hiden Analytical Ltd.) after separation and purification of argon.

## Electronic supplementary material

Supplementary Information

## References

[1] Marty B, Tolstikhin IN (1998). CO_2_ fluxes from mid-ocean ridges, arcs and plumes. Chem. Geol..

[2] Irwin WP, Barnes I (1980). Tectonic relations of carbon dioxide discharges and earthquakes. J. Geophys. Res..

[3] Marty B, Jambon A (1987). C/^3^He in volatile fluxes from the solid Earth: implication for carbon geodynamics. Earth Planet. Sci. Lett..

[4] Sano Y, Williams SN (1996). Fluxes of mantle and subducted carbon along convergent plate boundaries. Geophys. Res. Lett..

[5] Dasqupta R, Hirschmann MM (2006). Melting in the Earth’s deep upper mantle caused by carbon dioxide. Nature.

[6] Etiope G, Klusman RW (2002). Geologic emissions of methane to the atmosphere. Chemosphere.

[7] Etiope G, Lassey KR, Klusman RW, Boschi E (2008). Reappraisal of the fossil methane budget and related emission from geologic sources. Geophys. Res. Lett..

[8] Etiope, G. *Natural Gas Seepage*. *The Earth’s Hydrocarbon Degassing*. 213 pp, (Springer International Publishing, Switzerland, 2015).

[9] Kirschke S (2013). Three decades of global methane sources and sinks. Nature Geosci..

[10] IPCC. In *Climate Change2013: The Physical Science Basis*. *Contribution of Working Group I to the FifthAssessment Report of the Intergovernmental Panel on Climate Change*. (eds. Stocker, T.F. e*t al*.), 1535 pp., (Cambridge University Press, Cambridge, United Kingdom and New York, NY, USA, 2013).

[11] Kawagucci S (2013). Geochemical origin of hydrothermal fluid methane in sediment-associated fields and its relevance to the geographical distribution of whole hydrothermal circulation. Chem. Geol..

[12] Kietavainen R, Purkamo L (2015). The origin, source, and cycling of methane in deep crystalline rock biosphere. Front. Microbiol..

[13] Pinti DL, Gelinas Y, Moritz AM, Larocque M, Sano Y (2016). Anthropogenic and natural methane emissions from a shale gas exploration area of Quebec, Canada. Sci. Total Environ..

[14] Wakita H, Sano Y (1983). ^3^He/^4^He ratios in CH_4_-rich natural gases suggest magmatic origin. Nature.

[15] Etiope G, Sherwood Lollar B (2013). Abiotic methane on Earth. Rev. Geophy..

[16] D’ Alessandro, W. *et al*. Large compositional differences in the gases released from the Kizildag ophiolitic body (Turkey): Evidences of prevailingly abiogenic origin. *Marine and Petroleum Geology*. in press, 10.1016/j.marpetgeo.2016.12.017 (2017).

[17] Sano Y, Wakita H (1985). Geographical distribution of ^3^He/^4^He ratios in Japan: Implications for arc tectonics and incipient magmatism. J. Geophys. Res..

[18] Giggenbach WF, Sano Y, Wakita H (1993). Isotopic composition of helium, and CO_2_ and CH_4_ contents in gases produced along the New Zealand part of a convergent plate boundary. Geochim. Cosmochim. Acta.

[19] Italiano F, Yuce G, Uysal IT, Gasparon M, Morelli G (2014). Insights into mantle-type volatiles contribution from the dissolved gases in artesian waters of the Great Artesian Basin, Australia. Chemical Geology.

[20] Ring U (2016). Recent mantle degassing recorded by carbonic spring deposits along sinistral strike-slip faults, south-central Australia. Earth and Planetary Science Letters.

[21] Polyak BG (2000). Helium isotopes, tectonics and heat flow in the Northern Caucasus. Geochim. Cosmochim. Acta.

[22] Hilton, D. R., Fischer, T. P. & Marty, B. Noble gases and volatile recycling at subduction zones. in *Noble Gases in Geochemistry and Cosmochemistry*. (eds. Porcelli, D., Ballentine, C.J. & Wieler, R.), 319-370, (Mineralogical Soc. America, Washington, 2002).

[23] Sano, Y. & Fischer, T. P. The analysis and interpretation of noble gases in modern hydrothermal systems. In *The Noble Gases as Geochemical Tracers*. *Advances in* Isotope Geochemistry (ed. Burnard, P.), 249–317, (Springer-Verlag, 2013).

[24] Yuce, G. *et al*. Geochemical characteristics of soil radon and carbon dioxide within the Dead Sea Fault and Karasu Fault in the Amik Basin (Hatay), Turkey. *Chemical Geology*, in press, doi.org/10.1016/j.chemgeo.2017.01.003 (2017).

[25] Yuce G (2014). Origin and interactions of fluids circulating over the Amik Basin (Hatay-Turkey) and relationships with the hydrologic, geologic and tectonic settings. Chemical Geology.

[26] Wakita H, Sano Y, Urabe A, Nakamura Y (1990). Origin of methane-rich natural gas in Japan: formation of gas fields due to large-scale submarine volcanism. Appl. Geochem..

[27] Sakata S, Sano Y, Maekawa T, Igari SI (1997). Hydrogen and carbon isotopic composition of methane as evidence for biogenic origin of natural gases from the Green Tuff basin, Japan. Org. Geochem..

[28] Sano, Y., Marty, B. & Burnard, P. Noble gases in the atmosphere. in *The Noble Gases as Geochemical Tracers*. *Advances in Isotope Geochemistry* (ed. Burnard, P.), 17–31, (Springer-Verlag, 2013).

[29] Yang TF (2002). He-3/He-4 ratios of fluid samples in Taiwan. Geochim. Cosmochim. Acta.

[30] Yang TF, Chou CY, Chen C-H, Chyi LL, Jiang JH (2003). Exhalation of radon and its carrier gases in SW Taiwan. Rad. Meas..

[31] Yang TF (2005). Variations of soil radon and thoron concentrations in a fault zone aand prospective earthquakes in SW Taiwan. Rad. Meas..

[32] Zartman RE, Reynolds JH, Wasserburg GJ (1961). Helium, argon and carbon in some natural gases. J. Geophys. Res..

[33] Torgersen T, Kennedy BM (1999). Air-Xe enrichments in Elk Hills oil field gases: role of water in migration and storage. Earth Planet. Sci. Lett..

[34] Wen H-Y (2016). Helium and methane sources and fluxes of shallow submarine hydrothermal plumes near the Tokara Islands, Southern Japan. Sci. Rep..

[35] Bernard, B., Brooks, J. M. & Sackett, W. M. A geochemical model for characterization of hydrocarbon gas sources in marine sediments. Offsh. Technol. Conf., 2-5 May, Houston, Texas, 435–438 (1977).

[36] Whiticar MJ (1999). Carbon and hydrogen isotope systematics of bacterial formation and oxidation of methane. Chem. Geol..

[37] Coleman DD, Risatti JB, Schoell M (1981). Fractionation of carbon and hydrogen isotopes by methane-oxidizing bacteria. Geochim. Cosmochim. Acta.

[38] Schoell M (1983). Genetic characterization of natural gases. AAPG Bull..

[39] Moritz A (2015). Methane baseline concentrations and sources in shallow aquifers from the shale gas-prone region of the St. Lawrence Lowlands (Quebec, Canada). Environ. Sci. Technol..

[40] Etiope G (2009). Evidence of subsurface anaerobic biodegradation of hydrocarbons and potential secondary methanogenesis in terrestrial mud volcanoes. Mar. Petrol. Geol..

[41] Hong WL, Etiope G, Yang TF, Chang PY (2013). Methane flux from miniseepage in mud volcanoes of SW Taiwan: Comparison with the data from Italy, Romania, and Azerbaijan. J. Asian Earth Sci..

[42] Welhan JA, Lupton JE (1987). Light hydrocarbon gases in Guaymas basin hydrothermal fluids: thermogenic versus abiogenic origin. AAPG Bull..

[43] Charlou JL (1996). Mineral and gas chemistry of hydrothermal fluids on an ultrafast spreading ridge: East Pacific Rise, 17° to 19°S (Naudur cruise, 1993) phase separation processes controlled by volcanic and tectonic activity. J. Geophys. Res. Solid Earth.

[44] Pinti DL, Marty B (1995). Noble gases in crude oils from the Paris Basin, France: Implications for the origin of fluids and constraints on oil-water-gas interactions. Geochim. Cosmochim. Acta.

[45] Sherwood-Lollar B, Ballentine CJ, O’Nions RK (1997). The fate of mantle-derived carbon in a continental sedimentary basin: Integration of C/He relationships and stable isotope signatures. Geochim. Cosmochim. Acta.

[46] Sano Y, Marty B (1995). Origin of carbon in fumarolic gas from island arcs. Chem. Geol..

[47] Waseda A, Iwano H (2008). Characterization of natural gases in Japan based on molecular and carbon isotope compositions. Geofluids.

[48] Kawagucci, S. Fluid geochemistry of high-temperature hydrothermal fields in the Okinawa Trough. in *Subseafloor Biosphere Linked to Hydrothermal System: TAIGA Concept* (eds. Ishibashi, J.-i. *et al*.), 387-403, (Springer-Verlag, 2015).

[49] Yoshii T (1977). Crust and upper-mantle structure beneath northeastern Japan. Kagaku.

[50] Sun CH (2010). Origins of Taiwan’s mud volcanoes: Evidence from geochemistry. J. Asian Earth Sci..

[51] Hunt, M. J. *Petroleum Geochemistry and Geology*. (W.H. Freeman and Company, San Francisco, USA, 1979).

[52] Hoering TC, Moore HE (1958). The isotopic composition of the nitrogen in natural gases and associated crude oils. Geochim. Cosmochim. Acta.

[53] Stahl WJ (1977). Carbon and nitrogen isotopes in hydrocarbon research and exploration. Chem. Geol..

[54] Jenden PD, Kaplan IR, Poreda RJ, Craig H (1988). Origin of nitrogen-rich natural gases in the California Great Valley: evidence from helium, carbon and nitrogen isotope ratios. Geochim. Cosmochim. Acta.

[55] Murty SVS (1992). Noble gases and nitrogen in natural gases from Gujarat, India. Chem. Geol..

[56] Williams LB (1995). Nitrogen isotope geochemistry of organic matter and minerals during diagenesis and hydrocarbon migration. Geochim. Cosmochim. Acta.

[57] Zhu YN, Shi BQ, Fang CB (2000). The isotopic compositions of molecular nitrogen: implications on their origins in natural gas accumulations. Chem. Geol..

[58] Krooss BM (2005). Investigation of the pyrolytic liberation of molecular nitrogen from Palaeozoic sedimentary rocks. Int. J. Earth Sci..

[59] Mingram B, Hoth P, Luders V, Harlov D (2005). The significance of fixed ammonium in Palaeozoic sediments for the generation of nitrogen-rich natural gases in the North German Basin. Int. J. Earth Sci..

[60] Hoef, J. *Stable Isotope Geochemistry*. (Springer-Verlag, Berlin, Germany, 1980).

[61] Marty B, Humbert F (1997). Nitrogen and argon isotopes in oceanic basalts. Earth Planet. Sci. Lett..

[62] Cartigny P, Boyd SR, Harris JW, Javoy M (1997). Nitrogen isotopes in peridotitic diamonds from Fuxian, China: the mantle signature. Terra Nova.

[63] Sano Y, Takahata N, Nishio Y, Marty B (1998). Nitrogen recycling in subduction zones. Geophys. Res. Lett..

[64] Ooki (2016). Volatile element isotopes of submarine hydrothermal mineral deposits in the Western Pacific. Geochem. Geophys. Geosys..

[65] Roulleau E, Sano Y, Takahata N, Yang FT, Takahashi HA (2015). He, Ar, N and C isotope compositions in Tatun Volcanic Group (TVG), Taiwan: Evidence for an important contribution of pelagic carbonates in the magmatic source. J. Volcanol. Geother. Res..

[66] Kazaoka O (2015). Stratigraphy of the Kazusa Group, Boso Peninsula: An expanded and highly-resolved marine sedimentary record from the Lower and Middle Pleistocene of central Japan. Quat. Int..

[67] Matsuda T, Nakamura K, Sugimura A (1967). Late Cenozoic orogeny in Japan. Tectonophysics.

[68] You CF, Gieskes JM, Lee T, Yui TF, Chen HW (2004). Geochemistry of mud volcano fluids in the Taiwan accretionary prism. Appl. Geochem..

[69] Yang TF (2004). Composition and exhalation flux of gases from mud volcanoes in Taiwan. Environ. Geol..

[70] Kagoshima T (2016). Spatial and temporal variations of gas geochemistry at Mt. Ontake, Japan. J. Volcanol. Geother. Res..

[71] Takahata N, Nishio Y, Yoshida N, Sano Y (1998). Precise isotopic measurements of nitrogen at the sub-nanomole level. Anal. Sci..

[72] Schlitzer, R. Ocean Data View, http://odv.awi.de (2013). Date of access: April 23, 2017.

[73] Earth Observatory of Singapore, http://www.earthobservatory.sg/files/news/images/Tohoku2-bloc_diagramme_japan_earthquakes.jpg. Date of access: September 12, 2017.

