# Supplementary material for: Origin of methane-rich natural gas at the West Pacific convergent plate boundary
Source: Sci Rep. 2017 Nov 15;7:15646. doi: 10.1038/s41598-017-15959-5 (PMC5688071; doi:10.1038/s41598-017-15959-5)
Supplement: Supplementary file 1 — Supplementary Information [file 41598_2017_15959_MOESM1_ESM.pdf]

# Supplementary Information

## Origin of methane-rich natural gas at the West Pacific convergent plate boundary

Yuji Sano[1]*, Naoya Kinoshita[1], Takanori Kagoshima[1], Naoto Takahata[1], Susumu Sakata[2], Tomohiro Toki[3], Shinsuke Kawagucci[4], Amane Waseda[5], Tefang Lan[6], Hsinyi Wen[6,7], Ai-Ti Chen[6], Hsiaofen Lee[8], Tsanyao F. Yang[6#], Guodong[9] Zheng, Yama Tomonaga[10], Emilie Roulleau[11] and Daniele L. Pinti[12]

[1]Atmosphere and Ocean Research Institute, The University of Tokyo, Kashiwa, Japan.

[2]Institute for Geo-Resources and Environment, National Institute of Advanced Industrial Science and Technology, Tsukuba, Japan.

[3]Department of Chemistry, Biology and Marine Science, University of the Ryukyus, Okinawa, Japan.

[4]Department of Subsurface Geobiological Analysis and Research, Japan Agency for Marine-Earth Science and Technology, Yokosuka, Japan.

[5]Japan Petroleum Exploration Co., Ltd., Tokyo, Japan

[6]Department of Geosciences, National Taiwan University, Taipei, Taiwan.

[7]Green Energy and Environment Research Laboratories, Industrial Technology Research Institute, Taiwan.

[8]Institute of Earth Sciences, Academia Sinica, Taipei, Taiwan

[9]Key Laboratory of Petroleum Resources, Gansu Province / Key Laboratory of Petroleum Resources Research, Institute of Geology and Geophysics, Chinese Academy of Sciences, Lanzhou 730000, China.

[10]Institute of Geological Sciences, University of Bern, Bern, Switzerland.
[11]Laboratoire Magmas et Volcans, Université Clermont-Auvergne, CNRS - IRD, OPGC, 63178 Aubière, France
[12]GEOTOP & Départment des sciences de la Terre et de l'atmosphère, Université du Québec à Montréal, Montreal, Canada.

*: Corresponding author (e-mail: ysano@aori.u-tokyo.ac.jp)
[#]: Deceased

Table S1. Chemical compositions of natural gas samples.

| No. | Location | Name | $CH_4$ (%) | $C_2H_6$ (%) | $C_3H_8$ (%) | $CO_2$ (%) | $N_2$ (%) | $O_2$ (%) | Ar (%) | He (ppm) | Reference# |
|---|---|---|---|---|---|---|---|---|---|---|---|
| | *Frontal arc (South Kanto)* | | | | | | | | | | |
| 1 | South Kanto, Japan | Mobara-A1 | 99.0 | 0.04 | <0.01 | 0.70 | 0.31 | 0.01 | 0.007 | 7.0 | This work |
| 2 | South Kanto, Japan | Mobara-B1 | 98.6 | 0.04 | <0.01 | 1.06 | 0.42 | 0.01 | 0.008 | 9.0 | This work |
| 3 | South Kanto, Japan | Shirako-C3 | 96.1 | 0.03 | <0.01 | 3.49 | 0.56 | 0.01 | 0.011 | 17.0 | This work |
| 4 | South Kanto, Japan | Shirako | 98.9 | <0.01 | <0.01 | 0.72 | 0.42 | 0.01 | 0.005 | 0.7 | Wakita et al. (1990) |
| 5 | South Kanto, Japan | Yokoshiba-D1 | 99.1 | 0.02 | <0.01 | 0.70 | 0.28 | 0.01 | 0.005 | 7.0 | This work |
| 6 | South Kanto, Japan | Yokoshiba | 95.7 | 0.01 | <0.01 | 3.50 | 0.70 | 0.13 | 0.022 | 1.2 | Wakita et al. (1990) |
| 7 | South Kanto, Japan | Heiwajima | 97.6 | 0.01 | <0.01 | 1.20 | 1.54 | 0.08 | 0.028 | 3.4 | Wakita et al. (1990) |
| | *Volcanic-Back arc (Akita-Niigata)* | | | | | | | | | | |
| 8 | Tohoku, Japan | Akita, NR6 | 85.0 | 15.0* | | 0.01 | 0.60 | 0.01 | 0.006 | 14.0 | This work |
| 9 | Tohoku, Japan | Akita, AR80 | 84.2 | 15.8* | | 0.01 | 1.20 | 0.01 | 0.018 | 4.8 | This work |
| 10 | Tohoku, Japan | Akita, Yurihara1 | 86.4 | 7.40 | 3.80 | <0.01 | 0.60 | 0.04 | 0.002 | 23.0 | Wakita et al. (1990) |
| 11 | Tohoku, Japan | Akita, Yurihara2 | 74.2 | 11.4 | 8.76 | - | - | - | - | 12.0 | Sakata et al. (1997) |
| 12 | Tohoku, Japan | Akita, Yurihara3 | 82.0 | 10.7 | 4.96 | - | - | - | - | 9.0 | Sakata et al. (1997) |
| 13 | Tohoku, Japan | Akita, Kamihama | 80.9 | 1.68 | 0.19 | - | - | - | - | 110 | Sakata et al. (1997) |
| 14 | Tohoku, Japan | Yamagata, SR27 | 81.2 | 18.8* | | 0.01 | 0.20 | 0.01 | 0.003 | 2.7 | This work |
| 15 | Tohoku, Japan | Niigata, NS12 | 90.2 | 9.59* | | <0.01 | 0.10 | <0.01 | 0.002 | 285 | This work |
| 16 | Tohoku, Japan | Niigata, NS4 | 93.9 | 5.30* | | <0.01 | 0.42 | 0.11 | 0.004 | 167 | This work |
| 17 | Tohoku, Japan | Niigata, MG42 | 96.2 | 3.43* | | 0.17 | 0.07 | <0.01 | 0.002 | 231 | This work |
| 18 | Tohoku, Japan | Niigata, Shiunji | 86.0 | 7.77 | 3.34 | - | - | - | - | 5.0 | Sakata et al. (1997) |
| 19 | Tohoku, Japan | Niigata, Mitsuke | 92.3 | 4.77 | 1.40 | - | - | - | - | 34.0 | Sakata et al. (1997) |
| 20 | Tohoku, Japan | Niigata, Katakai | 83.0 | 4.81 | 1.65 | - | - | - | - | 32.0 | Sakata et al. (1997) |
| 21 | Tohoku, Japan | Niigata, Nakadori | 89.0 | 6.10 | 2.10 | <0.01 | 1.30 | 0.04 | 0.002 | 48.0 | Wakita et al. (1990) |
| 22 | Tohoku, Japan | Niigata, Hirai | 87.0 | 6.90 | 2.50 | <0.01 | 2.30 | 0.04 | 0.002 | 56.0 | Wakita et al. (1990) |
| | *Collision zone (Taiwan)* | | | | | | | | | | |
| 23 | South Taiwan | CL-1 | 16.7 | 0.10 | <0.01 | 75.1 | 11.2 | 0.01 | 0.029 | 51.3 | This work |
| 24 | South Taiwan | CL-2 | 16.0 | 0.14 | <0.01 | 85.3 | 7.28 | 0.01 | 0.010 | 29.9 | This work |
| 25 | South Taiwan | CL-3 | 24.4 | 0.38 | <0.01 | 65.3 | 13.9 | 0.02 | 0.082 | 156 | This work |
| 26 | South Taiwan | SYNH | 97.7 | 0.24 | <0.01 | 2.10 | 1.40 | 0.00 | 0.018 | 156 | This work |
| 27 | South Taiwan | WSD | 98.6 | 0.01 | <0.01 | 0.80 | 0.70 | 0.03 | 0.006 | 12.6 | This work |
| 28 | South Taiwan | SGS | 96.3 | 0.10 | <0.01 | 2.01 | 2.24 | 0.00 | 0.022 | 20.9 | This work |
| 29 | South Taiwan | SHTY | 91.8 | 4.26 | 1.25 | 1.86 | 1.23 | 0.01 | 0.029 | 142 | This work |
| 30 | South Taiwan | GSP | 94.8 | 0.13 | <0.01 | 4.37 | 0.98 | 0.00 | 0.010 | 33.0 | This work |
| 31 | South Taiwan | MP | 94.5 | 0.02 | <0.01 | 5.52 | 2.94 | 0.21 | 0.030 | 30.6 | This work |
| 32 | South Taiwan | LS | 83.4 | 0.17 | <0.01 | 0.08 | 21.0 | 1.28 | 0.109 | 15.3 | This work |
| 33 | South Taiwan | DingKun | 94.4 | 0.12 | <0.01 | 0.64 | 6.58 | 0.05 | 0.071 | 12.4 | This work |

#: There are much more data in Wakita et al.[21]. We selected typical samples.

*: Summation of $C_2H_6$ and $C_3H_8$.

"-": not measured

Table S2. Isotopic composition and abundance ratio of natural gas samples.

| No. | Name | $\delta^{13}C_{CH4}$ (‰) | $\delta^{15}N$ (‰) | $^3He/^4He$ (Ra) | $^4He/^{20}Ne$ | $^{40}Ar/^{36}Ar$ | $CH_4/^3He$ | $N_2/^{36}Ar$ | C1/C2+C3 |
|---|---|---|---|---|---|---|---|---|---|
| | *Frontal arc* | | | | | | | | |
| 1 | Mobara-A1 | -67.1 ± 0.3 | -0.7 ± 0.6 | 0.08 ± 0.05 | 36 | 304 ± 6 | 1.4E+12 | 1.3E+04 | 2370 |
| 2 | Mobara-B1 | -69.6 ± 0.4 | -0.4 ± 0.4 | 0.08 ± 0.03 | 33 | 304 ± 6 | 1.0E+12 | 1.5E+04 | 2300 |
| 3 | Shirako-C3 | -71.8 ± 0.4 | 0.2 ± 0.5 | 0.26 ± 0.01 | 85 | 298 ± 6 | 1.6E+11 | 1.5E+04 | 3360 |
| 4 | Shirako | -71.2 ± 0.2 | - | 0.24 ± 0.04 | 4.8 | - | 4.2E+12 | - | >20000 |
| 5 | Yokoshiba-D1 | -68.6 ± 0.4 | -1.1 ± 0.6 | 0.29 ± 0.07 | 26 | 295 ± 6 | 3.6E+11 | 1.5E+04 | 4410 |
| 6 | Yokoshiba | - | - | 0.25 ± 0.01 | 14 | - | 2.4E+12 | - | 9570 |
| 7 | Heiwajima | -74.6 ± 0.2 | - | 0.11 ± 0.01 | 100 | - | 1.9E+12 | - | 9760 |
| | *Volcanic-Back arc* | | | | | | | | |
| 8 | Akita, NR6 | -43.9 ± 0.2 | 1.5 ± 0.3 | 6.29 ± 0.14 | 2929 | 328 ± 10 | 6.9E+09 | 3.1E+04 | 5.68 |
| 9 | Akita, AR80 | -41.1 ± 0.3 | -0.7 ± 0.5 | 1.10 ± 0.04 | 2793 | 296 ± 9 | 1.2E+11 | 2.0E+04 | 5.35 |
| 10 | Akita, Yurihara1 | -37.6 ± 0.2 | - | 5.97 ± 0.18 | 625 | - | 4.5E+09 | - | 7.71 |
| 11 | Akita, Yurihara2 | -39.7 ± 0.2 | - | 6.83 | 112 | - | 6.5E+09 | - | 3.68 |
| 12 | Akita, Yurihara3 | -38.2 ± 0.2 | - | 6.63 | 266 | - | 9.9E+09 | - | 5.24 |
| 13 | Akita, Kamihama | -49.9 ± 0.2 | - | 7.38 | 1700 | - | 7.2E+08 | - | 43.3 |
| 14 | Yamagata, SR27 | -45.8 ± 0.3 | -0.6 ± 0.4 | 1.00 ± 0.03 | 623 | 287 ± 8 | 2.2E+11 | 1.7E+04 | 4.33 |
| 15 | Niigata, NS12 | -42.7 ± 0.2 | - | 2.81 ± 0.20 | 187 | 293 ± 13 | 8.1E+08 | - | 9.31 |
| 16 | Niigata, NS4 | -53.7 ± 0.2 | -2.3 ± 0.3 | 2.14 ± 0.15 | 14 | 284 ± 13 | 1.9E+09 | 3.0E+04 | 17.6 |
| 17 | Niigata, MG42 | -52.5 ± 0.2 | - | 1.85 ± 0.13 | 96 | 282 ± 12 | 1.6E+09 | - | 28 |
| 18 | Niigata, Shiunji | -37.7 ± 0.2 | - | 4.80 | 2500 | - | 2.6E+10 | - | 7.74 |
| 19 | Niigata, Mitsuke | -34.5 ± 0.2 | - | 7.66 | 59 | - | 2.6E+09 | - | 15 |
| 20 | Niigata, Katakai | -33.8 ± 0.2 | - | 7.79 | 142 | - | 2.4E+09 | - | 12.8 |
| 21 | Niigata, Nakadori | -33.1 ± 0.2 | - | 5.67 ± 0.18 | 2000 | - | 2.4E+09 | - | 10.9 |
| 22 | Niigata, Hirai | -33.0 ± 0.2 | - | 5.46 ± 0.11 | 830 | - | 2.1E+09 | - | 9.26 |
| | *Collision zone* | | | | | | | | |
| 23 | CL-1 | -35.9 ± 0.3 | 2.8 ± 0.5 | 4.88 ± 0.11 | 133 | 380 ± 8 | 4.8E+08 | 1.5E+05 | 162 |
| 24 | CL-2 | -46.8 ± 0.9 | 2.5 ± 0.4 | 5.95 ± 0.13 | 275 | 435 ± 9 | 6.5E+08 | 3.2E+05 | 118 |
| 25 | CL-3 | - | - | 4.07 ± 0.12 | 12 | 310 ± 7 | 2.8E+08 | 5.3E+04 | 64.2 |
| 26 | SYNH | -35.5 ± 0.3 | -1.7 ± 0.3 | 0.17 ± 0.05 | 167 | 319 ± 7 | 2.6E+10 | 2.4E+04 | 411 |
| 27 | WSD | -38.0 ± 0.3 | -4.2 ± 0.5 | 0.19 ± 0.07 | 36 | 402 ± 9 | 2.9E+11 | 4.8E+04 | 9860 |
| 28 | SGS | -44.1 ± 0.4 | -1.2 ± 0.3 | 0.16 ± 0.06 | 68 | 301 ± 7 | 2.1E+11 | 3.1E+04 | 988 |
| 29 | SHTY | -37.5 ± 0.3 | -1.1 ± 0.3 | 0.21 ± 0.03 | 135 | 319 ± 7 | 2.2E+10 | 1.4E+04 | 16.7 |
| 30 | GSP | -61.6 ± 0.3 | -2.2 ± 0.4 | 0.40 ± 0.05 | 626 | 343 ± 7 | 5.2E+10 | 3.4E+04 | 721 |
| 31 | MP | -36.8 ± 0.4 | -1.6 ± 0.3 | 0.06 ± 0.05 | 12 | 320 ± 7 | 3.9E+11 | 3.2E+04 | 5360 |
| 32 | LS | -36.9 ± 0.4 | 1.1 ± 0.4 | 1.41 ± 0.07 | 19 | 304 ± 7 | 2.8E+10 | 5.9E+04 | 481 |
| 33 | DingKun | -51.0 ± 0.3 | 0.5 ± 0.6 | 0.29 ± 0.10 | 25 | 302 ± 7 | 1.9E+11 | 2.8E+04 | 823 |

"-": not measured

Table S3. Deconvolution of methane in natural gas together with possible end members.

| | $\delta^{13}C_{CH4}$ (‰) | $CH_4/^3He$ | EPR (%) | Microbial (%) | Thermogenic (%) |
|---|---|---|---|---|---|
| *End members* | | | | | |
| EPR | -23 | 5.0E+06 | 100 | 0 | 0 |
| Microbial | -75 | 1.0E+13 | 0 | 100 | 0 |
| Thermogenic | -25 | 1.0E+13 | 0 | 0 | 100 |
| *Natural gas samples* | | | | | |
| Akita-Niigata | -53.7 ~ -33.0 | 7.2E+8 ~ 2.2E+11 | 0.21±0.05 | 32±3 | 68±3 |
| South Kanto | -74.6 ~ -67.1 | 1.6E+11 ~ 4.2E+12 | 0.0008 | 91±2 | 9.0±2.2 |
| South Taiwan | -61.6 ~ -35.5 | 2.8E+8 ~ 3.9E11 | 0.34±0.18 | 35±5 | 65±5 |
| Tokara | -29.7 ~ -24.8 | 2.9E+7 ~ 2.9E+9 | 3.2±1.8 | 6.3±1.2 | 91±2 |

Table S4. Geophysical data compilation in West Pacific convergent plate boundary.

| Geophysical data | South Kanto | Akita-Niigata | South Taiwan | Reference |
|---|---|---|---|---|
| Heat Flow | Low<br>30 ～ $40mW/m^2$ | High<br>60 ～ $70mW/m^2$ | High<br>50 ～ $70mW/m^2$ | Tanaka et al. [74]<br>Chi & Reed [75] |
| Seismic Velocity in crust<br>(P-wave perturvation) | Normal<br>1 ～ -1% | Slow<br>-1 ～ -2% | Slow<br>0 ～ -2% | Liu & Zhao [76]<br>Wu et al. [77] |
| Gravity Anomaly | Positive<br>50 ～ 150mGal | Negative<br>-50 ～ 20mGal | Normal<br>-20 ～ 0mGal | Ueda [78]<br>Yen & Yeh [79] |
| Crustal Deformation<br>(Horizontal displacement) | High<br>30mm/y | Low<br>10mm/y | High<br>40mm/y | Sagiya et al. [80]<br>Yu et al. [81] |
| Magnetic Anomaly | Negative<br>-80 ～ -100nT | Variable<br>-40 ～ +40nT | Normal<br>-20 ～ +10nT | Tanaka et al. [82]<br>Hsu et al. [83] |

Reference

[74] Tanaka, A., Yamano, M., Yano, Y & Sasada, M. Geothermal gradient and heat flow data in and around Japan (I): Appraisal of heat flow from geothermal gradient data. *Earth Planets Space* **56**, 1191-1194 (2004).

[75] Chi, W.-C. & Reed, D.L. Evolution of shallow, crustal thermal structure from subduction to collision: An example from Taiwan. *GSA Bulletin* **120**, 679-690 (2008)

[76] Liu, X. & Zhao, D. P and S wave tomography of Japan subduction zone from joint inversions of local and teleseismic travel times and surface-wave data. *Phys. Earth Planet. Int.* **252**, 1-22 (2016)

[77] Wu, Y.-M. et al. Seismic tomography of Taiwan: Imporoved constraints from a dense network of strong motion stations. *J. Geophys. Res.* **112**, B08312 (2007)

[78] Ueda, Y. Bouguer Gravity anomalies (ver. 2004) of Japanese Island Arcs and its adjacent area. *Rep. Hydro. Ocean. Res.* **41**, 1-26 (2005)

[79] Yen, H.-Y. & Yeh, Y.-H. Two-dimensional crustal structures of Taiwan from gravity data. *Tectonics* **17**, 104-111 (1998)

[80] Sagiya, T., Miyazaki, S. & Tada, T. Continuous GPS Array and Present-day Crustal Deformation of Japan. *Pure Appl. Geophys.* **157**, 2303-2322 (2000)

[81] Yu, S.-B., Chen, H.-Y. & Kuo, L.-C. Velocity field of GPS stations in the Taiwan area. *Tectonophys* **274**, 41-59, (1997)

[82] Tanaka, A., Okubo, Y. & Matsubayashi, O. Curie point depth based on spectrum analysis of the magnetic anomaly data in East and Southeast Asia. *Tectonophys.* **306**, 461-470 (1999)

[83] Hsu, S.-K., et al. New Gravity and Magnetic Anomaly Maps in the Taiwan-Luzon Region and Their Preliminary Interpretation. *TAO* **9**, 509-532.
